# Tangible symmetry elements and space-group models to guide from molecular to solid-state composition

**DOI:** 10.1107/S1600576721012218

**Published:** 2022-02-01

**Authors:** Nico Graw, Dietmar Stalke

**Affiliations:** a Institut für Anorganische Chemie, Georg-August-Universität, Tammannstraße 4, Göttingen, 37077, Germany

**Keywords:** space groups, education, symmetry elements

## Abstract

The ability to imagine symmetry and the spatial arrangement of atoms and molecules is crucial in chemistry in general. Teaching and understanding crystallography and the composition of the solid state therefore require understanding of symmetry elements and their relationships. To foster the student’s spatial imagination, models representing a range of concepts from individual rotation axes to complete space groups have been designed and built. These models are robust and large enough to be presented and operated in a lecture hall, and they enable students to translate conventional 2D notations into 3D objects and *vice versa*. Tackling them hands-on means understanding them.

## Abstract 2

Large and robust models of the most abundant space groups and symmetry elements are presented as an aid to visualization. In this way, space-group symmetry can be introduced to any audience in a readily understandable manner.

## 1. Introduction

Teaching and understanding symmetry are at the core of chemistry and crystallography. Very early on, chemistry students are exposed to the necessity of symmetry considerations in chemistry (Glasser, 1967 ▸; Jaffé, 2013 ▸). Typically, this is done with a focus on small molecules (McKay & Boone, 2001 ▸; Orchin & Jaffe, 1970 ▸; Zeldin, 1966 ▸), for example in the context of molecular syntheses (Brown, 1971 ▸; Caserio, 1980 ▸), or the implications of symmetry on spectroscopic properties (Carlos, 1968 ▸; Harris & Bertolucci, 2014 ▸; Myrick *et al.*, 2004 ▸).

Discussing molecular symmetry naturally requires spatial imagination. To gain and train this, various specific pre­fabricated and static molecular or structural models and general model kits can be very useful and are readily available (Birk & Foster, 1989 ▸; Flint, 2011 ▸; Fuchigami *et al.*, 2016 ▸; Scalfani & Vaid, 2014 ▸; Sein, 2010 ▸). At this stage, the Schönflies notation is usually introduced to the students and used to describe symmetry elements and point groups. Transition from single molecules to crystalline matter often takes place at a much later point during studies, so that the previously learned framework in which to describe symmetry has to be extended. The concept of translation then requires the introduction of the Herman–Mauguin notation. From our teaching experience, students struggle with this, specifically with translational symmetry elements like screw axes and glide planes. Ultimately this also causes problems understanding crystallographic space groups and their depictions filed in *International Tables for Crystallography* (Brock *et al.*, 2006–2016 ▸), Volume A.

In our experience most students find space groups challenging to imagine, although they might cope with molecular symmetry. That spatial imagination is linked to student success rates, not only in crystallography or chemistry but in the natural sciences in general, has already been shown (Wai *et al.*, 2009 ▸). In addition, there is evidence that teaching using haptic and tactile feedback improves students’ ability for visuospatial thinking (Wu & Shah, 2004 ▸; Jones *et al.*, 2006 ▸). In the context of the natural sciences, it has been shown that tangible 3D objects have an improved effectiveness compared with 2D representations (Höst *et al.*, 2013 ▸; Moeck *et al.*, 2014*a* ▸) or virtual 3D models (Herman *et al.*, 2006 ▸) when they are integrated into teaching. This necessitates, of course, that suitable models are available. Due to the high degree of specialization, these often need to be custom made, for which 3D printing is a powerful method. 3D-printed models have been applied in the teaching of biomolecular self-assembly (Olson *et al.*, 2007 ▸), single-crystal X-ray crystallography (Brannon *et al.*, 2020 ▸), materials science and engineering (Rodenbough *et al.*, 2015 ▸), periodicity and aperiodicity (Casas, 2020 ▸), point groups (Casas & Estop, 2015 ▸), and molecular symmetry (Brown *et al.*, 2019 ▸). This is facilitated by the fact that structural data can be retrieved from open access databases and converted into printable formats using freely available software (Moeck *et al.*, 2014*b* ▸; Kaminsky *et al.*, 2014 ▸). However, even though a great number of creative unit-cell models for all kinds of teaching purposes have been reported (Bindel, 2002 ▸; Cady, 1997 ▸; Elsworth *et al.*, 2017 ▸; Kennard, 1979 ▸; Komuro & Sone, 1961 ▸; Kildahl *et al.*, 1986 ▸; Laing, 1997 ▸; Lenzer *et al.*, 2019 ▸; Li & Worrell, 1989 ▸; Ma *et al.*, 2020 ▸; Mann, 1973 ▸; Mattson, 2000 ▸; Olsen, 1967 ▸; Scattergood, 1937 ▸; Sein & Sein, 2015 ▸; Seymour, 1938 ▸; Sunderland, 2014 ▸; Westbrook & DeVries, 1957 ▸), to the best of our knowledge no models have been made available so far to foster students’ abilities in spatial imagination in the contexts of translational symmetry and space groups.

## 2. Axes of rotation

To demonstrate rotational symmetry of any given object about an axis of rotation, in principle no model is needed. Just arranging the correct number of copies of the object on a surface would be sufficient to provide the correct representation. However, we wanted to have a model that enables this simple rotational symmetry demonstration to be performed in a vertical manner so that its materialization and operation can be easily observed by an audience of students in a lecture hall. Correct and incorrect can easily be demonstrated, even with chiral objects. The model also facilitates introducing the students to the symbolic representations typically used to depict axes of rotation of *n*th order.

Models for two-, three-, four- and sixfold rotational symmetry were manufactured by cutting the respective shapes from 10 mm black coated aluminium. Each piece was fitted with a steel rod at the back to be clamped in a commercial laboratory stand. Small holes equipped with lock screws were drilled in the sides of the aluminium shapes according to the symmetry represented.

**Figure 1:**
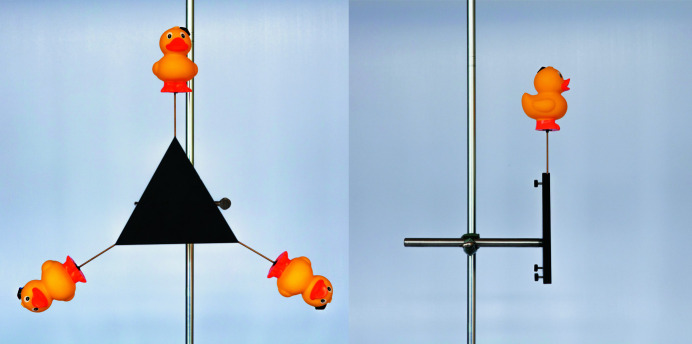
Models of a threefold rotational axis. (Left) Front view as it would be perceived by the audience. (Right) Side view showing only one position populated and the lock screws for securing the rubber ducks.

As objects to perform the symmetry operations we chose rubber ducks because they are available in a huge variety of colours and shapes (even chiral and achiral ones) and are expected to catch the students’ interest. These rubber ducks were fixed on sturdy metal wire, which can be inserted into the predrilled holes and secured in place by tightening the lock screws. In this way the rubber ducks can be added step by step, even in a horizontal setup (Fig. 1 ▸, and Figs. S1 and S2 in the supporting information).

When slightly unclamped from the laboratory stand, the whole model can be rotated manually to give a visual demonstration of the indistinguishable configuration of rubber ducks resulting from executing the symmetry operation. Misarrangements might be introduced deliberately and corrected later to emphasize the unique arrangement governed by the rotational axis.

## 3. Screw axes

**Figure 2:**
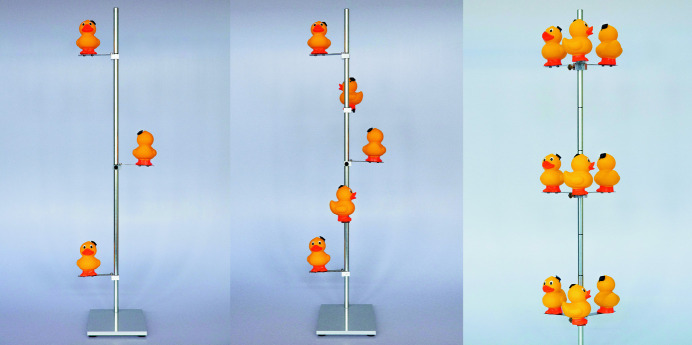
(Left) A model of a 2_1_ screw axis. (Middle) A model of a 4_1_ screw axis. (Right) A model of a 6_3_ screw axis.

Proceeding from the rotational axes to screw axes, a set of objects can no longer be arranged in a plane perpendicular to the viewing direction. Having the rotation performed in a vertical manner as described above no longer carries any advantage. We therefore based the model solely on a standard laboratory stand. To keep the freedom of assembling the model by adding rubber ducks step by step, we constructed a mount which allows the rubber ducks to be moved along the metal rod and to be rotated around it. These mounts were made from blocks of aluminium, with a central hole to fit onto the laboratory stand and a locking screw. The rubber ducks were fixed on pieces of sheet metal strong enough to hold their weight without bending, while the sheet metal itself was screwed to the aluminium block. This ensemble allows the rubber ducks to be arranged freely around the laboratory stand at any height (Fig. 2 ▸, and Figs. S3–S6 in the supporting information). The freedom is provided to generate any screw-axis symmetry if the appropriate number of rubber ducks is arranged accordingly.

For better differentiation between *e.g.* 3_1_ and 3_2_ or the various 4 and 6 types of screw axes it might be helpful to extend the model to the length of two cell edges (Cockcroft & Driessen, 2009 ▸; Grenier & Ballou, 2012 ▸). The design of the screw-axis models allows one to add as many rubber ducks on one laboratory stand as needed. As they do not carry explicit labelling, their height may correspond to any multiple of a cell edge depending on how it is used by the instructor.

In the cases of 4_2_, 6_2_, 6_3_ and 6_4_ screw axes, where two or three objects take the same position with respect to the direction of translation, special mounts were made to hold several rubber ducks simultaneously so that they can be placed at the same height along the laboratory stand. To assist with the placing of the rubber ducks, evenly spaced indentations were made on the laboratory stands. Finally, in order to incorporate the official symbols used by *International Tables for Crystallography* to depict screw axes, the top of the laboratory stand can be flagged with the appropriate symbols.

## 4. Space-group models

In a final step we set out to construct complete models of space groups. While a space group describes an infinite array of unit cells, for practical reasons we decided to build models that depict only one unit cell. Besides being accurate space-group representations, the models were designed to fulfil two additional requirements. Firstly, the model should be constructed in a way that easily allows it to be looked at from different perspectives. In particular, if viewed along one of the crystallographic axes the model’s appearance should generate an impression that visually resembles the depiction of that space group in *International Tables for Crystallography*. In this way, a direct connection between 2D projection and 3D arrangement can be drawn for chosen examples, thus helping students to gain a general understanding of how to read space-group depictions. Hence the models needed to be maximally transparent. This also fulfils three of the five principles suggested for the design of chemistry visualization tools, namely offering multiple representations, promoting transformation between two and three dimensions, and visualization of linked referential connections (Wu & Shah, 2004 ▸).

The second requirement was for the model itself to be a representation of the space group’s symmetry elements inside a unit cell only. If it was necessary for reasons of stability of the overall construction, additional transparent elements were added for rigidity. Having only an assembly of symmetry elements allows the teacher to emphasize that neither cell contents nor metrics but only the symmetry elements and their relative arrangement determine crystal classes and ultimately space groups (Brock & Lingafelter, 1980 ▸). Additionally, a model without cell contents also gives the students the opportunity to add these contents themselves. Starting with an object placed on any general position, students are able to perform symmetry operations by hand when adding additional objects at the required positions. This offers a hands-on experience of how symmetry elements act on an asymmetric unit, and it emphasizes the limitations present in the asymmetric units in combination with certain symmetry elements (Brock & Lingafelter, 1980 ▸).

**Figure 3:**
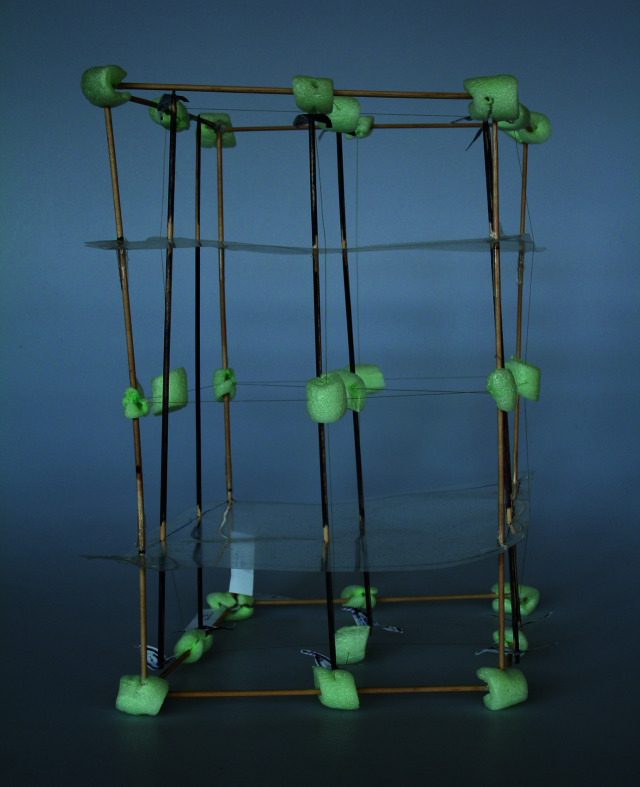
A prototype model of space group *P*2_1_/*c* made from scrap materials. The view is along the crystallographic **c** direction.

We decided to build models of space groups 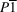 , *P*2_1_ and *P*2_1_/*c*. With these the complexity of the overall symmetry can be increased stepwise. We started by making small-scale models (cell edges between 10 and 20 cm) which were extremely helpful to show how all the different components come together to form the entire space group. As these models were too small to allow the easy arrangement of objects inside, and probably not sturdy enough to have them handled by several students, large-scale models were also built. However, if one only seeks a 3D representation of a space group, these small-scale models might very well be suitable and can be made at reasonably low cost. The only materials used were wooden skewers, polystyrene packaging beads, thread, paper, overhead transparencies and a hot glue gun (Fig. 3 ▸, and Fig. S7 in the supporting information). Alternatively, we have prepared a set of object files, which are included in the supporting information, that can be 3D printed to construct a small model of *P*2_1_ (Fig. S8 in the supporting information).

The robust large-scale models (cell edges between 40 and 60 cm) were constructed in our faculty’s workshop. To keep the models lightweight, 4 or 6 mm aluminium rods were used to build the screw axes and cell edges when needed. To distinguish between them the screw axes were painted black. Inversion centres – modelled as solid spheres – and the symbols for the 2_1_ screw axes were 3D printed from black poly(lactic acid) filament. In both cases they were custom designed using the freely available *Tinkercad* 3D modelling program (https://www.tinkercad.com) with preformed and aligned holes to accommodate the aluminium rods. This was especially handy in the cases of 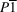 and *P*2_1_/*c*, where an inversion centre is located on every corner of the unit cell. With these adapted holes in the printed spheres, they serve as connectors and aid in maintaining the correct angles throughout the model. Glide planes were made from plates of polyacrylate cut into shape and covered with strips of transparent coloured foil. The orientation of these coloured strips was chosen so that they align with the direction of the translational component of the glide plane. Additional parts that were needed solely for reasons of stability (*e.g.* to fix an inversion centre in the centre of a face of the unit cell) were made from colourless transparent polyacrylate rods or plates.

**Figure 4:**
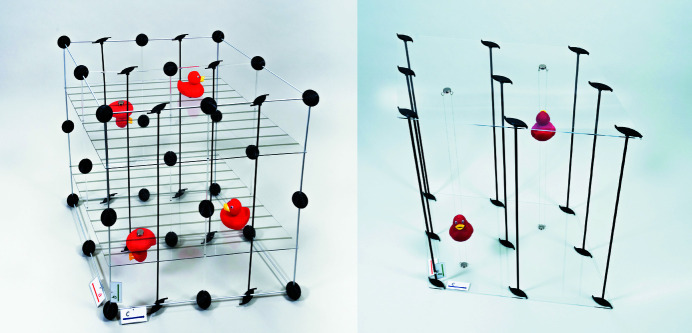
(Left) A model of space group *P*2_1_/*c*. Red ducks represent all general positions within one unit cell (*Z* = 4). (Right) A model of space group *P*2_1_. Red ducks represent all general positions within one unit cell (*Z* = 2).

In the case of *P*2_1_/*c*, ducks can easily be put on top of or beneath the glide planes if magnets are used to keep them in place (Fig. 4 ▸). This also allows them to be positioned and moved freely with respect to the glide plane. Because the models of space groups 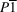 and *P*2_1_ do not contain any solid planes within the unit cell, we devised two options to populate the asymmetric unit. For the first option, one of the ducks is placed at the bottom of the model. A stand in transparent polyacrylate was constructed to hold a second duck at the appropriate height and orientation given by applying the respective symmetry operation. The second option is to suspend the ducks on strings which then can be stretched across the model with hooks and magnets (Fig. 4 ▸). In the latter case the ducks can in principle be placed at any position within the unit cell, while the stands do not grant this freedom. However, the stand can certainly be handled more easily.

**Figure 5:**
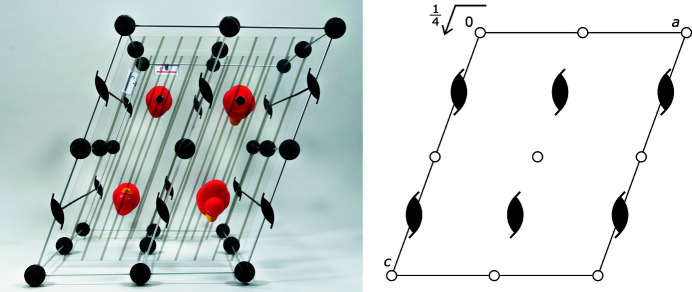
(Left) A model of space group *P*2_1_/*c*, viewed along the crystallographic **b** direction. (Right) A schematic depiction of space group *P*2_1_/*c*, viewed along the crystallographic *b* direction.

The option to place the ducks – or any other symbolic unit-cell content – freely inside the model, and therefore also the choice to take them out completely with only the symmetry elements left, enables the model to accomplish the aforementioned second condition. Once the models are presented along certain crystallographic directions (Fig. 5 ▸, and Figs. S9–S12 in the supporting information), they clearly resemble the space-group depictions in *International Tables for Crystallography*. In particular, they are helpful to understand how the symmetry elements represented in the 2D depiction also translate into the third direction perpendicular to the plane shown.

In the same vein, the models can also help the student to learn how to deal with fractional coordinates. Referring to the example shown in Fig. 5 ▸, students do not need just to imagine the glide plane at height ¼*b* as the model can easily be rotated, unlike the depiction, and be viewed perpendicular to the **b** direction. Additionally, this approach makes it immediately clear why there has to be a second glide plane within the unit cell at height ¾*b*, which of course is generated by symmetry but often forgotten by students just looking at the depiction. The same holds true for every other symmetry element that is not located within the depicted plane.

## 5. Conclusion

We have designed and built 3D models that represent individual symmetry elements and complete space groups. These models are large and robust enough to be presented in a lecture hall or classroom setting. They are made from sturdy materials so that they can be handled by students. All models allow for step-by-step assembly regarding the objects on which symmetry operations are performed. Tackling them hands-on means understanding them.

## Supplementary Material

Additional figures. DOI: 10.1107/S1600576721012218/gj5276sup1.pdf

3D construction files. DOI: 10.1107/S1600576721012218/gj5276sup2.bin
