# Supplementary material for: Tangible symmetry elements and space-group models to guide from molecular to solid-state composition
Source: J Appl Crystallogr. 2022 Feb 1;55(Pt 1):144–8. doi: 10.1107/S1600576721012218 (PMC8805161; doi:10.1107/S1600576721012218)
Supplement: Supplementary file 1 [file j-55-00144-sup1.pdf]

JOURNAL OF
APPLIED
CRYSTALLOGRAPHY

Volume 55 (2022)

Supporting information for article:

Tangible symmetry elements and space-group models to guide from molecular to solid-state composition

Nico Graw and Dietmar Stalke

S1. Models of axes of rotation

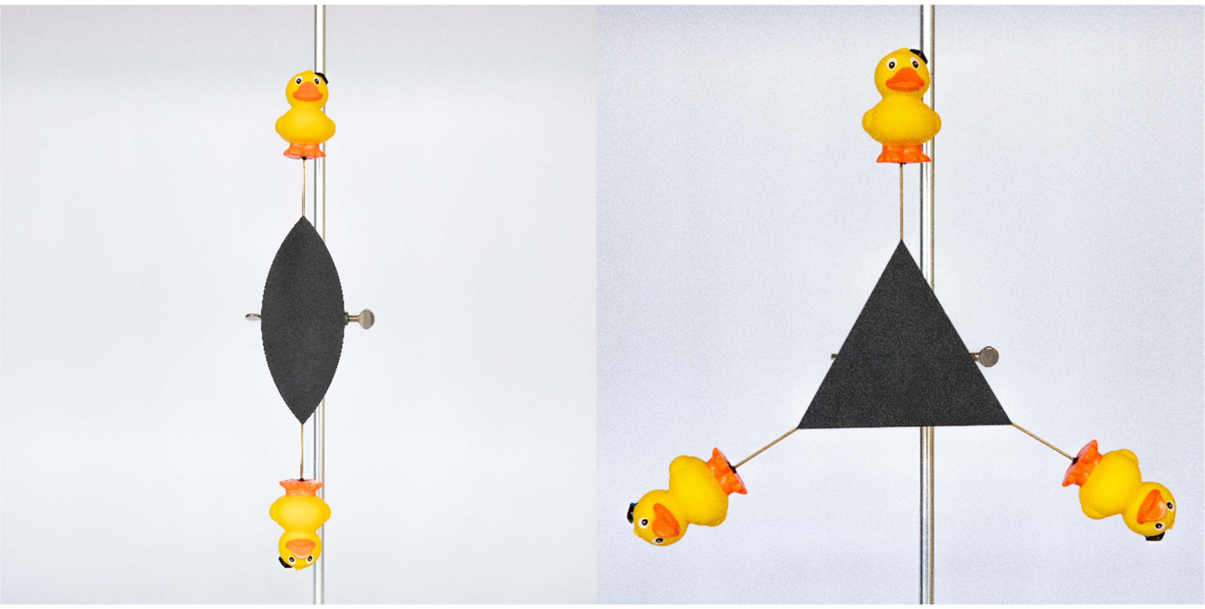

Figure S1 Models of rotational axes. Left: two-fold rotational axis. Right: three-fold rotational axis.

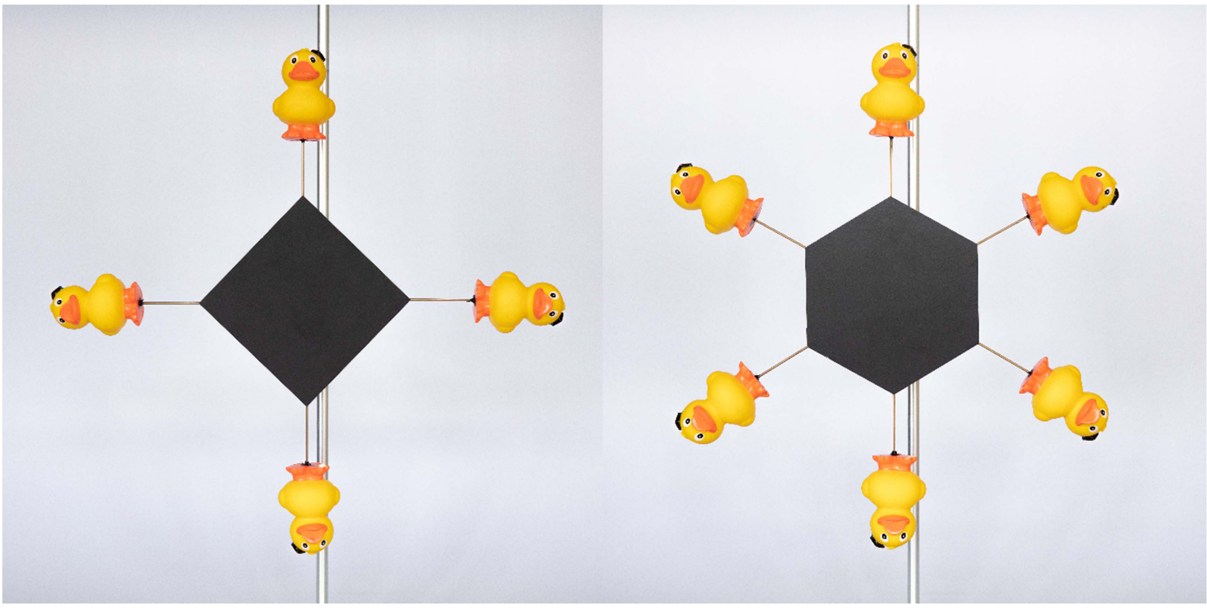

Figure S2 Models of rotational axes. Left: four-fold rotational axis. Right: six-fold rotational axis.

S2. Models of screw axes

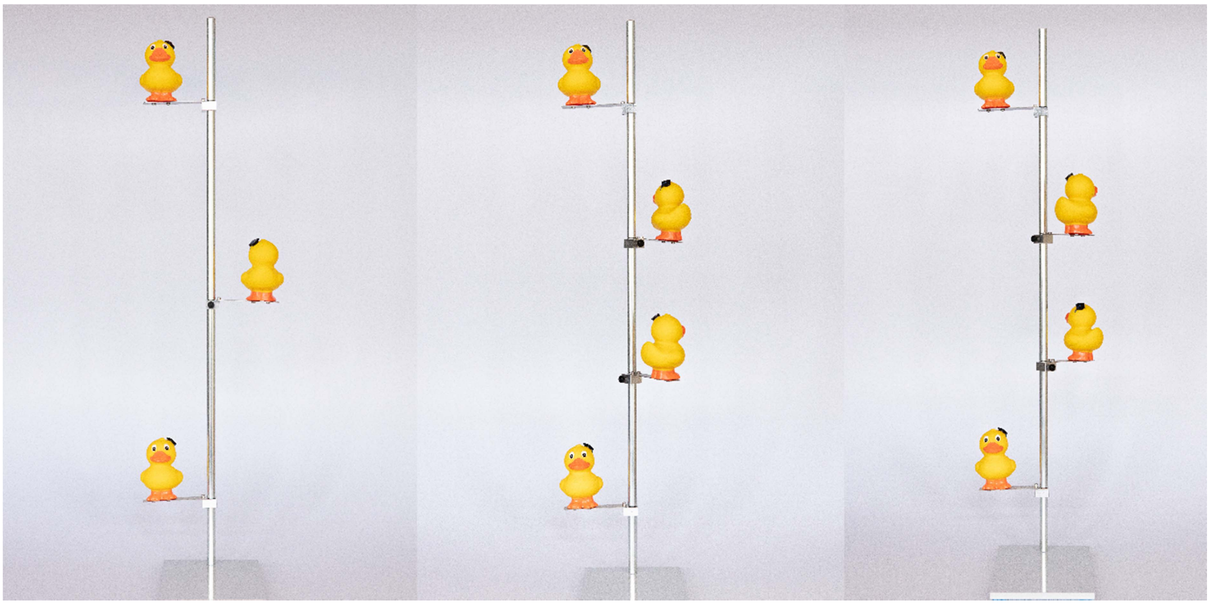

Figure S3 Models of screw axes. Left: 2_1 axis. Middle: 3_1 axis. Right: 3_2 axis.

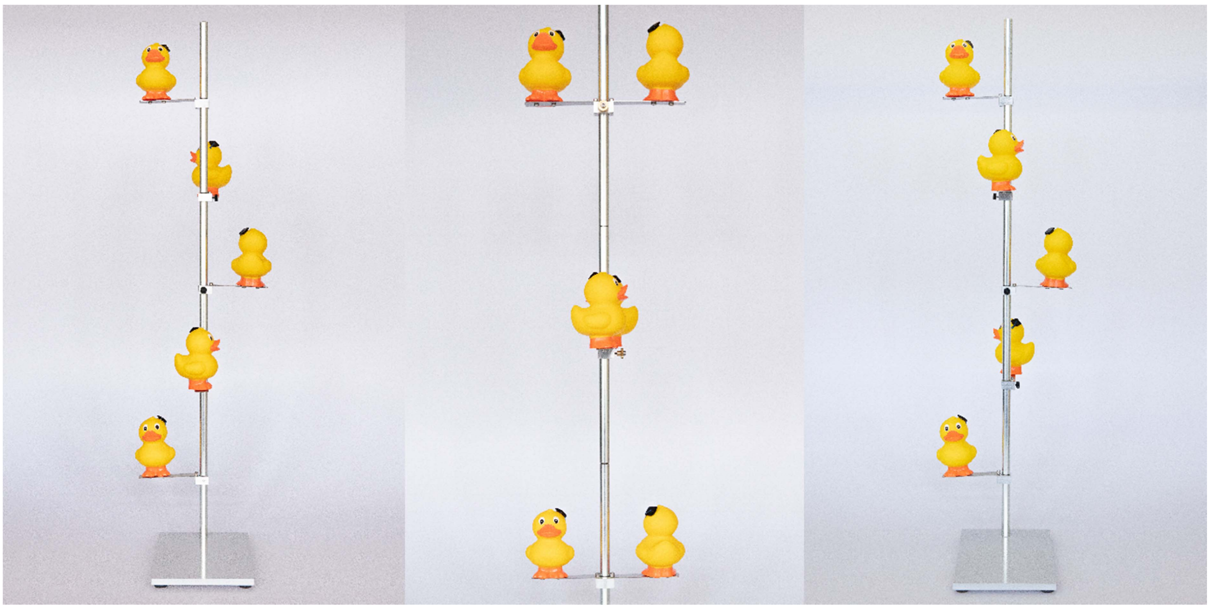

Figure S4 Models of screw axes. Left: 4_1 axis. Middle: 4_2 axis. Right: 4_3 axis.

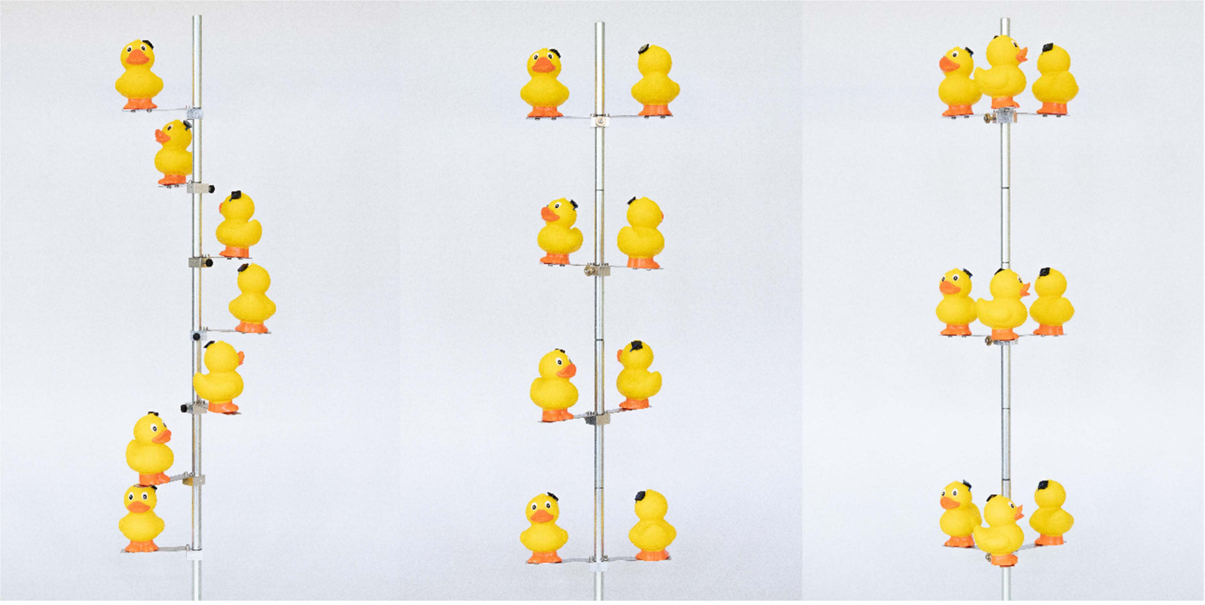

Figure S5 Models of screw axes. Left: 6_1 axis. Middle: 6_2 axis. Right: 6_3 axis.

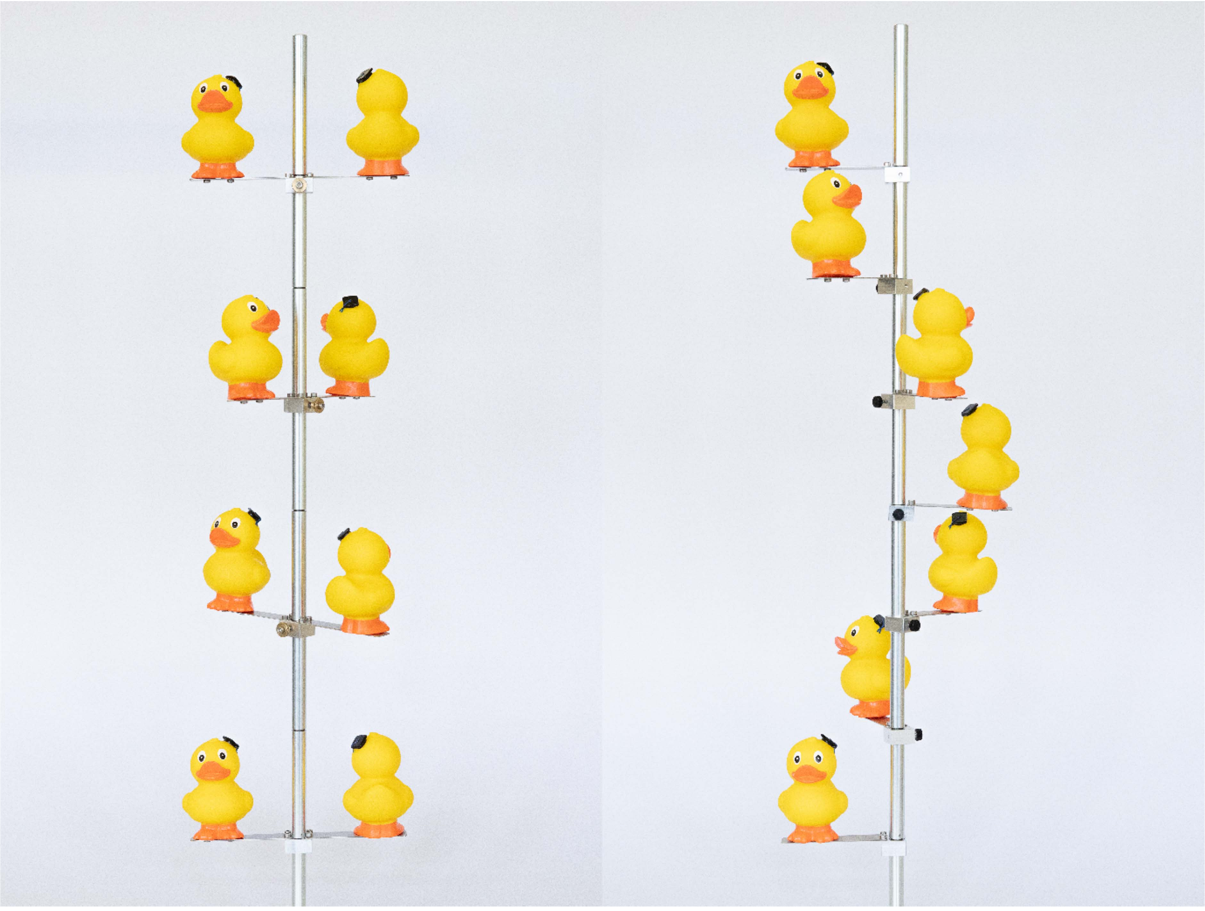

Figure S6 Models of screw axes. Left: 6_4 axis. Right: 6_5 axis.

S3. Models of space groups $P2_1$ and $P2_1/c$

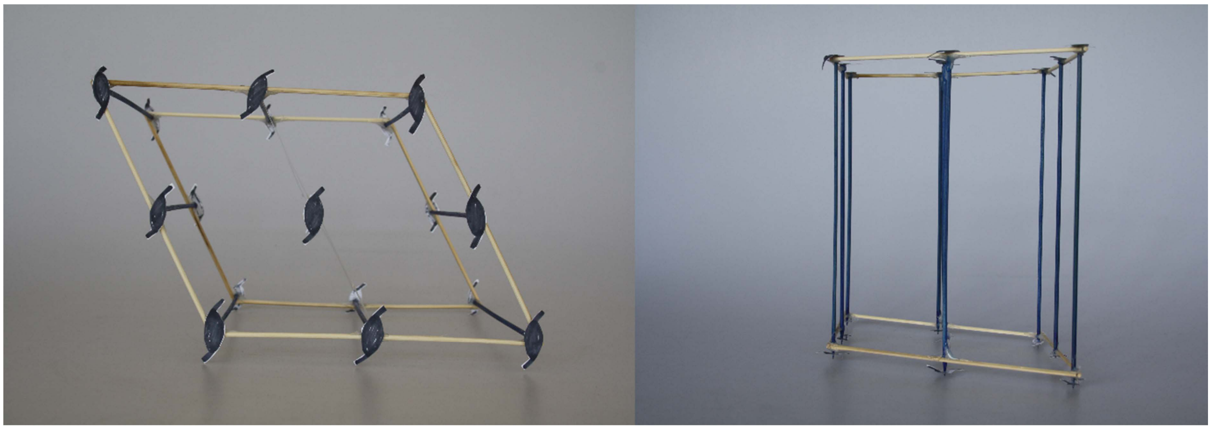

Figure S7 Prototype model of space group $P2_1$ made from scrap material. Left: View along crystallographic b direction. Right: View along crystallographic a direction.

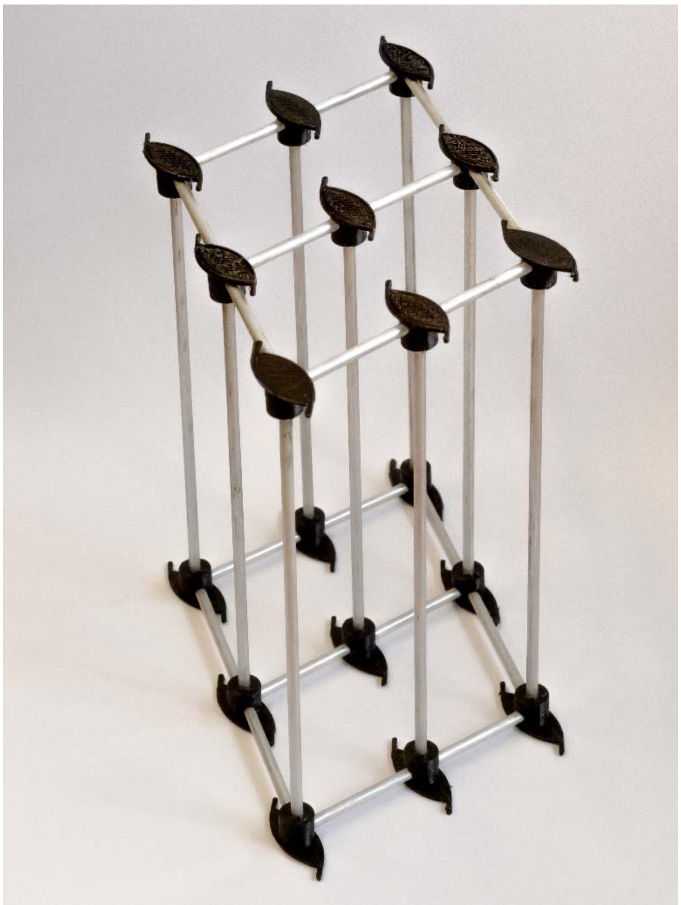

Figure S8 Small scale model of space group $P2_1$.

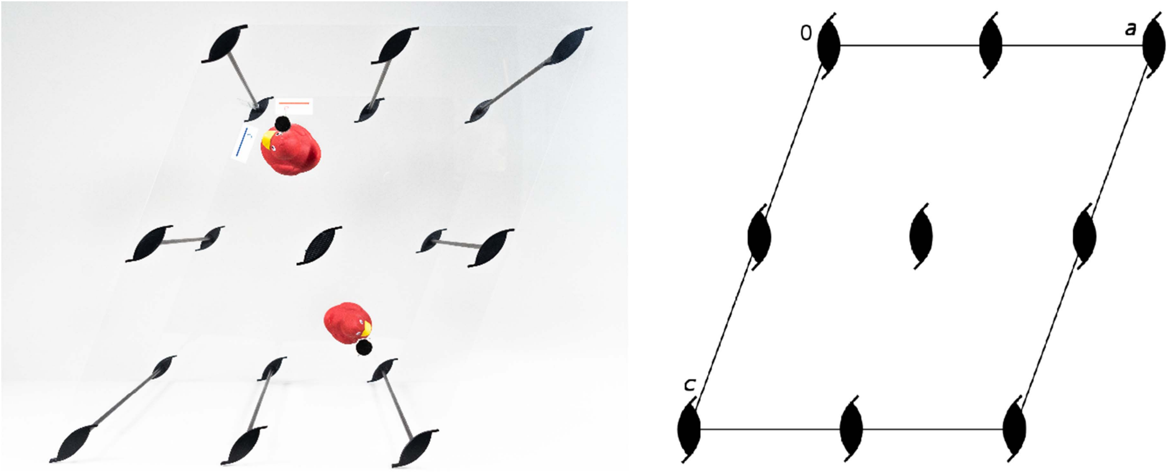

Figure S9 Left: Model of space group $P2_1$. View along crystallographic b direction. Right: Schematic depiction of space group $P2_1$. View along crystallographic b direction.

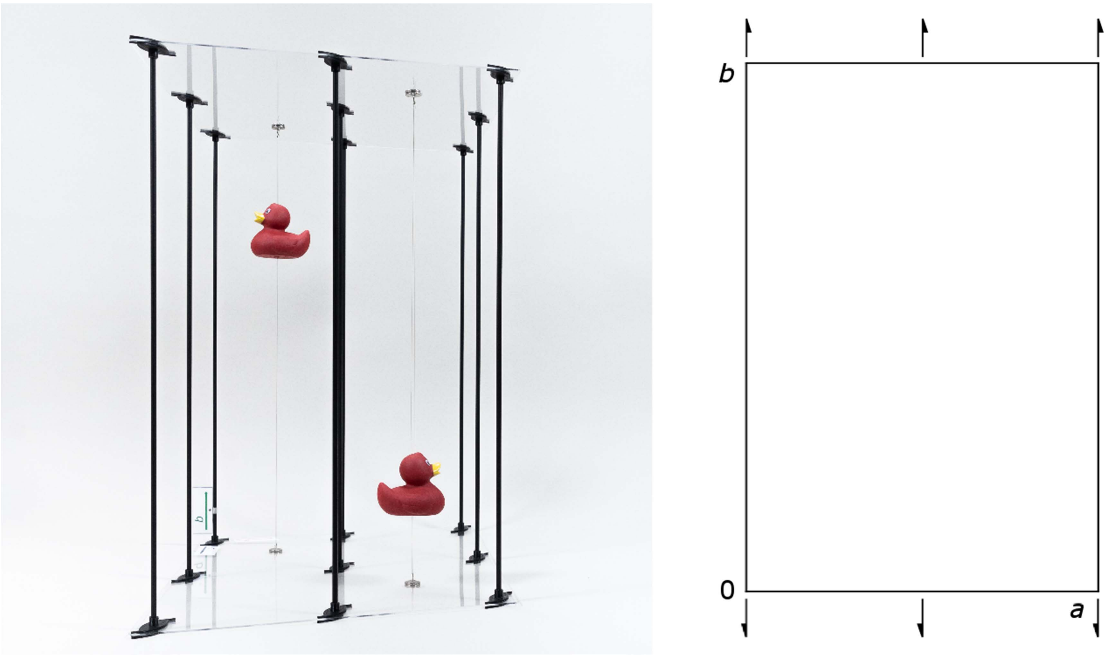

Figure S10 Left: Model of space group $P2_1$. View along crystallographic c direction. Right: Schematic depiction of space group $P2_1$. View along crystallographic c direction.

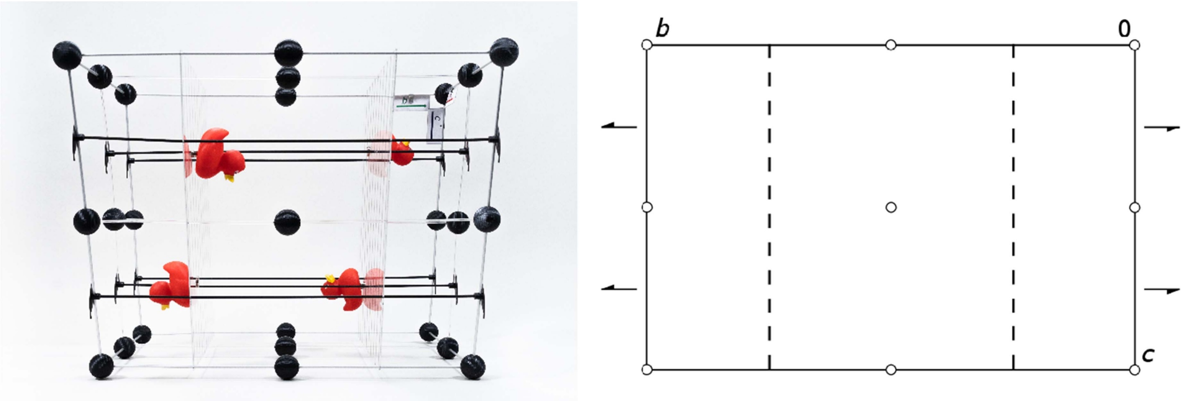

Figure S11 Left: Model of space group $P2_1/c$. View along crystallographic a direction. Right: Schematic depiction of space group $P2_1/c$. View along crystallographic a direction.

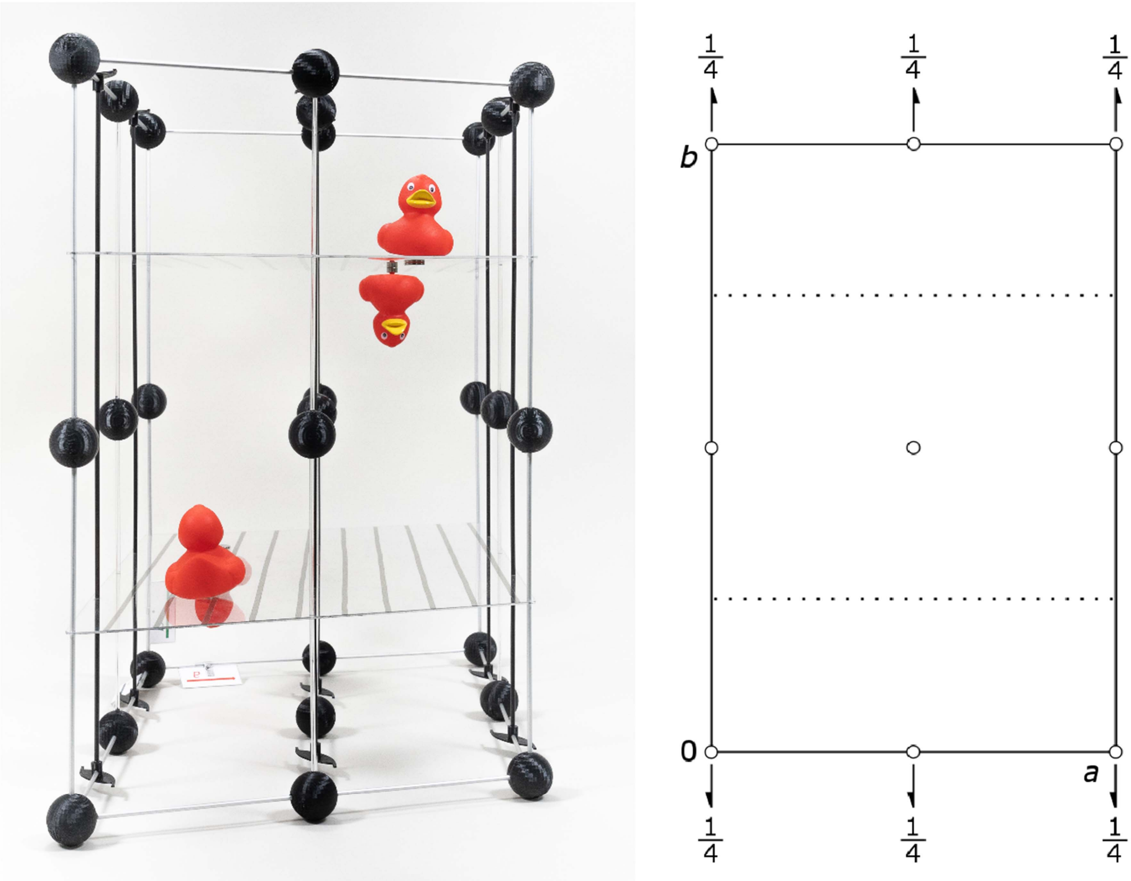

Figure S12 Left: Model of space group $P2_1/c$. View along crystallographic c direction. Right: Schematic depiction of space group $P2_1/c$. View along crystallographic c direction.
